# From Coaching to Neurocoaching: A Neuroscientific Approach during a Coaching Session to Assess the Relational Dynamics between Coach and Coachee—A Pilot Study

**DOI:** 10.3390/bs13070596

**Published:** 2023-07-16

**Authors:** Riccardo Valesi, Giorgio Gabrielli, Margherita Zito, Mara Bellati, Marco Bilucaglia, Alessia Caponetto, Alessandro Fici, Annarita Galanto, Massimiliano Giuseppe Falcone, Vincenzo Russo

**Affiliations:** 1 Department of Management, University of Bergamo, 24129 Bergamo, Italy; riccardo.valesi@unibg.it; 2 Department of Business, Law, Economics and Consumer Behaviour “Carlo A. Ricciardi”, Università IULM, 20143 Milan, Italy; giorgio.gabrielli@iulm.it (G.G.); margherita.zito@iulm.it (M.Z.); alessandro.fici@iulm.it (A.F.); vincenzo.russo@iulm.it (V.R.); 3 Behavior and Brain Lab IULM—Neuromarketing Research Center, Università IULM, 20143 Milan, Italy; bellatimara@gmail.com (M.B.); master.neuromarketing@iulm.it (A.C.); 4 Skillmatch-Insubria Group, Università Carlo Cattaneo—LIUC, 21053 Castellanza, Italy; agalanto@liuc.it; 5 Connect4Climate, The World Bank Group, Washington, DC 20433, USA; gfalcone@worldbankgroup.org

**Keywords:** coaching, neurocoaching, midlife transitions, professional development, neurosciences, helping relationship

## Abstract

Life transitions represent moments characterized by changes that can profoundly influence individual life trajectories and subjective well-being. Recently, career coaching has become an important method of helping people expand their self-awareness, facilitate personal development, and increase their performance in the school-to-work transition. Although previous studies have confirmed that one of the most important keys to the success of a coaching program is the quality of the relationship between coach and coachee, there is a lack of knowledge regarding how to objectively measure it. In this pilot study, we adopted a neuroscientific approach to introduce objective measures of the relationship between coach and coachee through the phases of a coaching session. A sample of 14 university students and a professional coach participated in career-coaching sessions while their affective states were measured by recording brain (EEG) and physiological (Skin conductance) activity. Electroencephalographic indicators of valence, arousal, and engagement showed differences between session phases, highlighting the possibility of a neurophysiological measurement of relational dynamics. Our results provide initial evidence that neurophysiological activity can be considered a way to understand differences in the coach-coachee relationship, thereby providing information on the effectiveness of coaching interventions and facilitating a better life transition from school to work.

## 1. Introduction

### 1.1. The Origins of Coaching

The definition of coaching has been applied in several fields, such as sport, life, and business. Even though coaching was first used in sport as a discipline that helps athletes maximize their performances, there is an increasing number of coaches who work for every kind of company as executive, team, or business coaches. Nowadays, brands like IBM and Motorola offer coaching programs as part of their business development plans [1,2]. The International Coach Federation (ICF), indeed, estimates about 47,500 business coaches around the world [3], and a great number of Fortune 500 companies use coaches to work with their managers [4]. There are many definitions of coaching. John Whitmore [5] is surely one of the pioneers of coaching; he created the GROW (goals, reality, options, will) method, which is a sort of guide that highlights the questions that a good coach should ask coachees. He published the book *Coaching for Performance*, suggesting that coaching is about “unlocking a person’s potential to maximize their own performance. It is helping them (clients) to learn rather than teaching them”. Laura Whitworth and Thomas Leonard, on the other hand, define coaching as the “relationship of possibilities […] based on trust” [6]. Stelter [7], again, offers another point of view about individual coaching and describes it as “the coach’s participation in the development and learning process of the person in focus. This process creates the foundation for new, alternative, or revised narratives of the focus person’s personal and professional life”. Coaching is not therapy, or counseling. It is an ongoing, participative, empowering, and collaborative method [8]. The first association that brings together coaches in the whole world is the International Coach Federation (ICF), founded in 1995. In the educational field, it was in Sidney in 2002 that some specialized courses and a master’s degree were introduced.

### 1.2. The Relationship between Coach and Coachee and Differences with Other Professionals

It is necessary to outline the difference between coaching and other types of helping relationships, such as mentoring, advising, and career counseling. The mentor is usually a senior employee who works for the same company as the individual he is trying to help. The latter is typically a younger employee with no experience in the field [9,10]. Their relationship, which can last even 5 years [10,11,12,13], is very close and based on trust. On the other hand, the coach is often an external worker, and the relationship between him and the coachees is not that long and close. It is based on improving coachees’ skills and capabilities. The adviser is characterized by high business acumen and technical expertise; it suggests to the client how to cope with operational issues and plan strategic actions [10,14]. The coach does not tell the coachee what to do or give business recommendations [10,15], he suggests how to accomplish great results by improving oneself. Eventually, the career counselor is someone who helps people highlight their job preferences before they start working or before changing jobs. The coach, instead, usually tries to help people who already work for a company improve their skills [10,16]. Although these coach profiles are not the only ones involved in helping relationships, they are of particular importance in the personal development of students at different stages of life [10,17].

Over the past two decades, the academic literature has offered a high number of articles focused on describing coaching, its evaluation methods, and how coaches can affect coachees’ lives. Nowadays, the efficacy of coaching is well established, and this is the reason why researchers started investigating the “so-called active ingredients of successful coaching—in particular the nature of the coach-coachee relationship and its impact on coaching effectiveness” [18,19,20]. In spite of this, there is still a great lack of scientific studies that focus on coaching and the relationship between coach and coachee, which is actually the essential condition for the success of coaching [21]. Indeed, it is known that the connection between coach and coachee has a high impact on coaching outcomes [22,23]. De Haan [19] reported, for example, the coach qualities valued by coachees, such as listening, understanding, and encouraging. O’Broin and Palmer [24] conducted research demonstrating that bond, engagement, collaboration, attitudes, and characteristics of the coach were assessed by both coaches and coachees as the most important aspects in relation to the creation of a coaching relationship. In addition, coaching focuses on dialogues: the coach is a sort of facilitator of the dialogue; “he or she is aware of the risk of inadvertently influencing the process of co-creation” [7,25]. In this dialogue, there are some key aspects between coach and coachee, such as trust, consciousness, responsibility, and freedom [5]. It is known that “coach’s behaviours, dispositions, education, and experiences are determinants of coaching success” [26,27,28]. Every coach has to listen to the coachee’s experiences, choices, desires, and aims without judging. The coachee, instead, has to be honest and motivated to achieve his/her goals [5].

### 1.3. From One to Many—The Difference between One-to-One, Group, and Team Coaching

Nowadays, it is possible to see an increasing shift from one-to-one coaching to group or team coaching. Each type of coaching has different pros and cons. One-to-one coaching “enables clients to dig deep into key focus areas” through the application of core competencies. It allows the coach and the coachee to design their relationship agreement, build a sense of trust and intimacy, improve communication and awareness, and focus on powerful conversations [29,30]. Team coaching can be defined as “a process that involves the collaboration of its members through the integration of different skills and perspectives” [31]. Team coaching has been considered helpful to “improve performance, and the processes by which performance is achieved, through reflection and dialogue” [32,33]. The shift from one-to-one to group or team coaching allowed the possibility to work on multiple agendas, facilitate teamwork, and focus on a wider scope. The benefit of this type of coaching is that it lets teams achieve greater results by working together and focusing on the common goal of the organization they work for. However, there are many voices and points of view, so it is not always possible to go deep into conversations [29,33].

### 1.4. The Advantages of Coaching in Relation to the Type of Clients

Coaching is for anyone: parents, adolescents, entrepreneurs, and athletes who want to improve their lives through a learning process [34]. It has different advantages depending on the type of client. In the business field, for example, it aims at individuals, managers, or organizations. Gilley and Gilley ([35], p. 6) suggested that the main benefits of coaching in the company relate to different dimensions:

- a more positive self-perception (individual dimension), in terms of enhanced relationship with manager, self-esteem, job satisfaction, career development, attention to the individual, individual and professional growth;
- improved interpersonal skills (managerial dimension), in terms of enhanced relationship with employees, focus on worker’s strengths and weaknesses, worker’s motivation and performance, soft skills development, career opportunities;
- higher overall organizational performance (organizational dimension) in terms of reduced complaints and lawsuits, enhanced general communication, working performance, teamwork and competitiveness, development of organizational culture and learning.

Coaching perks are also related to students in the educational field and individuals who are experiencing life transitions. Research conducted in 2016, for example, showed that “educational coaching is effective on students’ academic motivation, error-oriented motivation, and educational stress […] Educational coaching increases the students’ academic motivation” [36,37].

### 1.5. Coaching Methods: Kinesiology Coaching and Core Coaching

There are many methods used to conduct coaching sessions; in this paper, we aim to focus on kinesiology coaching during life transitions and the role of Core Coaching. Core Coaching was born about 30 years ago from the idea of Wolfgang Stabentheiner, creator of the FUTURE method and founder of FUTURE—Training Beratung Coaching (Innsbruck, Austria). The aim of the discipline is to respond to an increasingly widespread need among human beings: to achieve a greater awareness of one’s own way of being, of living, of dealing with everyday life, of relating to others, of finding meaning in one’s own existence, and of the unique and precious contribution that each of us is called upon to make. Core Coaching is a method in continuous evolution, just as our existence and the times we go through. Core has its origins in the most ancient oriental philosophies, in Gestalt, in Rogers’ client-centered approach, in the Jungian principle of individuation, in the systemic approach, and in humanistic psychology from Maslow onwards. Further important suggestions come from Assagioli’s psychosynthesis, transpersonal psychology, Montessori’s pedagogy, and the Phyllis Krystal Method, which makes use of simple symbols used in a specific way within processes that we could define as repetitive. The procedure of the Phyllis Krystal Method is similar to that of a guided meditation and is based on work linked to active imagination [38] and autogenous formation [39]. The use of symbols and processes consists of activating harmonization or transformation of impulses in the unconscious in order to increase personal autonomy and authenticity. Wolfgang Stabentheiner, in creating his method, has done a great job of integration, selecting the best of each of these approaches and acting on three levels/perceptual planes: listening to content (cognitive level), energy resonance (emotional level), and direct feedback of the body (physical level) through the kinesiological muscle test. The kinesiological test is an investigative tool; it is a muscle test that Dr. George Goodheart, discoverer of this technique, has defined as “the code to speak to the body and receive answers from it: the body does not lie, but we must ask the right question in the right way” [40,41,42,43,44]. Each Core Coaching session introduces us to an intense inner journey, which starts with the choice of a session theme and then, through a series of questions (chosen with the help of the Kinesiological test), brings out pieces of existence, memories, pain, emotions, and successes as parts of a single puzzle, which gradually goes to composition, giving the coachee great clarity and deep awareness. All this then helps to trigger a powerful transformative process that focuses on concrete actions to achieve challenging, regenerating, and functional objectives for a satisfying and meaningful existence. The questions tested, chosen, and used during the session can be classified into three main categories and relate to each of the three phases of a Core Coaching session: 1) “Identification questions”, which help to bring out what will be part of a subsequent re-elaboration; 2) “Transformative questions”, which help to trigger the process of reworking and transformation in the coachee; 3) “Integration questions”, which help to close the open cycles in the first two phases. In this last phase, the most immediate effect of a conscious closure of a cycle is that of immediate well-being, great centrality and focus on the objectives set at the start of the session, as well as the ability to contain dysfunctional and dispersive dynamics typical of open cycles [45,46,47]. Therefore, according to the instructions provided by the core-coaching professional involved in the study, each coaching session is structured in three phases:

1. A first contact and knowledge phase, in which the coach begins to develop the relationship with the coachee and identifies the goals that need to be achieved;
2. A second phase where the coachee deepens his or her experiences, emotions, and cognitions to explore issues that hinder the achievement of goals, identify solutions to the problems, and gain greater self-awareness;
3. A third and last phase where the coachee gained self-awareness and a greater understanding of the problem and its solution thanks to the guidance provided by the coach.

### 1.6. Life Transitions

Expected or disruptive and stressful significant changes within the life course can be defined as life transitions [48,49]. These changes may affect several aspects of life, such as relationship trajectories, educational trajectories, employment, physical health trajectories, and consumption patterns [50,51,52,53,54,55,56,57,58]. As highlighted in the report by the New Economics Foundation (NEF) for the UK Department of Food and Rural Affairs, life transitions can be considered “moments of change” [59,60], described as events involving major lifestyle changes and the disruption of behavioral patterns [61,62]. The succession of these changes often involves a redefinition of behavior through a process consisting of several stages. Various pieces of evidence have shown that the probability of changing and adapting behavior following a transition event is higher if the events are consistent with the stage the subject is in [63,64]. According to this, several studies have highlighted that behavioral changes following important life events are strongly connected to subjective interpretations of life transitions. Subjective interpretation makes it possible to give meaning to the event [65,66], develop interpretative skills [67,68], and produce effects that affect not only the transition period but also the internal course of life and individual personality [69,70,71]. Bauer and McAdams [72], for instance, highlighted how the interpretation of life transition in terms of growth has an impact not only on the satisfaction of the transition but also on generic personality development. Although life transitions affect the entire life course, it is possible to identify critical periods in which events seem to have greater relevance [49,73]. Among them, it is possible to consider childhood transition [74] with a focus on the role of educators, relatives, and educational transitions [75,76,77]. Another wide area of research focused on midlife transitions. These transitions have often been analyzed, focusing on critical events such as the transition to parenthood [78]. In later life, it is possible to mention critical periods such as retirement and widowhood [79,80]. If retirement is a very delicate period in the reorganization of energies and time previously dedicated to work [81], widowhood is considered one of the most distressing moments of a later age [79,80,82,83]. Although the thresholds for self-assessment and perception of age are increasingly variable and subjective later in life [84], one of the main factors connected with greater self-assessments of health and lifestyle in this period seems to be the active engagement in leisure activities [85,86,87]. The theoretical framework of our research, however, is related to one of the most explored areas of research in life transition, which concerns young adults and the transition to adulthood [88,89]. The life transition to adulthood includes events such as leaving home, considered a fundamental step towards increasing autonomy and developing the adult personality [48,90], and the school-to-work transition. The first concerns leaving the original household and is influenced by a wide number of variables, although this is earlier and more likely in industrialized countries [91,92]. Several studies have highlighted that gender [93,94], cultural background [95], family relationships and family resources [96,97,98,99] can influence this crucial event. The second concerns the transition from school to work. Ng e Feldman [100] defined it as the “first major adjustment” for young adults and conceptualized it with the notion of school-to-work transition (STWT). In their work, the authors identified this period as fundamental to setting individuals patterns related to work abilities, sense of self-efficacy, and decision-making, with consequent implications for organizations.

Considering the delicacy of the transitional moment and the important role of coaching in empowering and guiding individuals in their life choices, coaching methods can be a key component in facing transitions. Coaching techniques and models have been applied during life transitions in order to support people at a particular moment of their lives, to be able to face future life transitions, and to enhance personal resources, such as resilience, to strengthen life abilities, including after the COVID-19 pandemic as a well-being practice [8,101,102,103].

Recently, the advent of neuroscience in various disciplines, such as consumer neuroscience [104], neuromanagement [105], and neuropsychotherapy [106], has proven to provide valuable contributions in understanding both the physiological mechanisms underlying psychic processes and how the mind and body can influence each other. These new techniques have also started to be integrated into the field of coaching [107,108] and promise to be a new line of research in the years to come.

### 1.7. Neurocoaching: A Neuroscientific Approach to Coaching

A formal definition of the field of neurocoaching has been introduced by the International Coaching Council, according to which neurocoaching can be considered the fusion of cognitive neuroscience, neuropsychology, cognitive neuroplasticity, and cognitive behavioral therapy techniques [109]. Specifically, neurocoaching refers to the use of methods, techniques, and knowledge related to the study of the brain and human psychophysiology as supporting elements in the diagnosis, intervention, and evaluation of coaching practices [110,111]. As pointed out by some authors [112], it is only recently that findings from neuroscience have begun to be integrated into coaching applications [107,108], and consequently, this means that there are few research studies aimed at investigating the neural basis involved in coaching. Although the literature on neurocoaching is in its early days, the research conducted so far is particularly interesting. Matte and van Otterdijk [113] used EEG, surface electromyogram (SEMG), skin conductance (SC) [114], peripheral temperature, blood volume pulse (BVP), and respiration rate (RR) on some coachees to compare their psychophysiological data before and after a coaching intervention. The purpose of the study was to use the biofeedback method to improve the subjects’ ability to self-regulate their internal states, thereby increasing their well-being and self-efficacy. The results demonstrated the effectiveness of the coaching program in generating a reduction in stress symptoms, as evidenced by a decrease in sympathetic nervous system activity observed in the form of a reduction in masseter muscle tension and a level of skin conductance activation. Jack and colleagues [115] applied fMRI to test the brain responses associated with two coaching styles called Positive Emotional Attractor (PEA) and Negative Emotional Attractor (NEA): the former is characterized by positive emotional states [116] fostered by the coach’s ability to feel compassion for the individual’s hopes and dreams [115]. The latter is based on negative emotional states [116], in which the coach focuses on external criteria for success and acts on the coachee’s weaknesses [115]. PEA style was shown to be linked to brain regions such as the lateral occipital cortex, superior temporal cortex, medial parietal, subgenual cingulate, nucleus accumbens, and left lateral prefrontal cortex. Some of these brain areas are involved in visioning or perceptual imagery (i.e., the creation of an imagined event such as goals, objectives, and outcomes) [117,118,119], compassion [120], and social engagement [121]. In addition, in PEA emerged the activation of the parasympathetic nervous system, which is associated with the mechanisms of stress reduction, increased cognition, and positive emotions [122,123]. Finally, this style elicited positive emotions through activation of approach motivation measured by prefrontal asymmetry [124,125,126]. Instead, NEA-style activation activates medial prefrontal regions and the right lateral prefrontal cortex, which are related to the sympathetic nervous system and are implicated in stress response [127]. Moreover, NEA stimulated negative affects through the activation of avoidance motivation, estimated in terms of prefrontal asymmetry [125].

A further study conducted by Passarelli and colleagues [128] with fMRI explored the PEA and NEA coaching types in relation to information processing styles called global attention and local attention: the first focuses on the whole and promotes creative, global thinking and the integration of new, uncertain, or incomplete data into inclusive, superordinate knowledge structures. The second emphasizes differentiation, attention to detail, and the use of narrow cognitive categories that may lead to the omission of some incoming information [129,130]. The results showed that the PEA style produced activation of regions of visual cortex in the right hemisphere associated with imagination and global attention, while the NEA style elicited activation of regions of primary visual cortex in the left hemisphere, which are instead involved in processing basic visual sensory details and local attention [131,132]. Fingelkurts and colleagues [133] adopted the technique of quantitative electroencephalography (qEEG), consisting of an EEG measurement combined with automatic mathematical-statistical analyses. According to a “neuro-screening” logic, the authors identified nine qEEG metrics related to cognitive, emotional, and motivational functions, with which they defined the specific psychological profile of each coachee. Specifically, the metrics used were called vigilance, speed of cognitive and memory performance, internal attention, emotional-motivation tendency, sociability, anxiety tendency, stress resistance and recovery, overall brain resources, and deviation from optimal brain state. Based on this psychophysiological profile, the coach can customize the coaching program in order to enhance the effectiveness of the interventions. To demonstrate the usefulness of neurocoaching, the authors compared the qEEG profiles before and after each coaching intervention, showing a reduction in negative psychological states and an increase in personal well-being. Indeed, the results of the study showed that the coaching program was able to improve seven metrics, except internal attention, stress resistance, and recovery.

Puspa and colleagues [134] found that when a coach helps a coachee set new personal goals and a plan to achieve them, on the one hand, there is an increase in delta activity in the left prefrontal, frontal, central parietal, and occipital regions, and on the other hand, there is an increase in beta-gamma activity in the frontal, posterior temporal, and occipital areas. The delta and beta-gamma bands have been linked to the two motivational mechanisms of wanting and liking, respectively [135,136]. Wanting refers to the motivation to achieve a rewarding result. Liking indicates the imagined anticipation of the hedonic state of pleasure resulting from achieving the goal [134,137,138,139].

The research team led by Heyns-Nell [140] measured the power of the alpha band and event-related potentials (ERPs) N170 and P300, indicators of cortical arousal and engagement, in association with coaching effectiveness. In fact, previous pioneering studies have shown that arousal and cortical engagement are correlated with greater cognitive resources [135,141,142,143] and more effective decision-making [144,145]. The study demonstrated that the coaching interventions produced an increase in these neurological indicators, signaling an improvement in the coachees’ decision-making ability [140]. A subsequent study carried out by Bartolomé and colleagues [146] compared the effectiveness of directive and non-directive coaching styles in enhancing the coachee’s ability to develop creative insights (i.e., divergent thinking), considered an effect of strengthening problem-solving skills. The non-directive coaching style showed greater activation of the alpha and theta bands in the right temporal region and alpha, theta, and gamma in the right parietal area. Such brain activations, which are associated with processes of creative ideation [147,148,149,150], metacognition [151,152], and problem solving [153,154], provided neurophysiological evidence that coaching can enhance insight-based problem-solving skills [146].

Despite the studies described above, the research strand of neurocoaching is still in its early stages and needs further research. In this regard, a new topic that has not yet been investigated concerns the relational dynamics between coach and coachee during an actual coaching session.

Therefore, the pilot study we conducted is to be considered exploratory and is aimed at addressing certain aspects not yet considered by the current neurocoaching literature. First, the neuroscientific studies of neurocoaching have investigated neuropsychophysiological states elicited during a coaching session, but this paper specifically focuses on the relational dimension involving both coach and coachee. The importance of the construct of relational bonding is due to the fact that, although there are many variables to measure the effectiveness of coaching interventions [155], various evidence suggests that one of the key criteria for coaching success relies on the relationship between coach and coachee [24,156,157,158,159]. In turn, one of the main aspects underlying an effective social relationship is the emotional dimension of the people involved [160,161]. We therefore chose to use four neurophysiological indicators that the neuroscientific literature has shown to be correlated with particular properties of emotions and that have previously been applied to the analysis of relational dynamics: Approach-Withdrawal Index (AWI) [162,163,164,165], Beta over alpha ratio (BAR) [166,167], Beta over alpha plus theta ratio (BATR) [168], and Skin Conductance Level (SCL) [114,169,170,171,172]. Research has shown that AWI can be considered an indicator of emotional valence, BAR and SCL of arousal, and BATR of engagement. Additionally, such psychophysiological states have been used to measure properties of relational dynamics and social perception. In fact, valence has been utilized to estimate the emotional quality experienced by two interacting individuals based on the members’ ability to be relationally responsive [165]; arousal for the level of involvement in therapeutic sessions to measure empathic attunement [169,170,171,172]; and engagement for the detection of prosocial and empathic tendencies towards individuals perceived as similar to oneself [173].

These indices were measured in our study on both coach and coachee and were computed according to what we propose as a measure of “relational similarity” based on the Dynamic Time Warping (DTW) technique, a shape-based distance measure very popular in the literature of time series distance measures that find the best similarity of two signals applying a temporal warping (i.e., contraction or dilatation) [174,175]. Indeed, mechanisms of emotional resonance and synchronized bioelectric patterns have previously been observed during social interactions, especially those that produce a kind of “bonding” between subjects [176]. Second, there are no studies that have investigated the process dimension of a real coaching session by comparing its phases. We therefore identified a coaching approach called Core Coaching, whose sessions have a structure based on three consequential phases in which the dialogue between coach and coachee is continuously measured by neurophysiological devices. For each of these phases, neurophysiological indices were estimated. Third, ours is the first research that applies neuroscientific techniques to study the coaching approach of Core Coaching. The literature on coaching has identified several criteria by which to classify approaches that foster personal and professional growth. Among these criteria, it is possible to consider the theoretical model (e.g., psychodynamic, cognitive behavioral, person-centered, and narrative) [177], the number of the coachees (i.e., individual/one-to-one coaching and team coaching) [29], and the nature of the coachee (e.g., executive coaching, business coaching, and career coaching) [16,178,179]. In this regard, a professional coach adopting the Core Coaching approach was chosen for the study, as this approach is used in career coaching. In turn, career coaching represents the type of coaching on which the following research is based. In addition, we utilized a general approach rather than a specific coaching technique because it has been shown that it is the general orientation or approach to coaching that elicits important changes in the coachee [157]. Finally, there are no previous neurocoaching studies that have investigated that particular phase of life development involving the transition from the young adult stage to the adulthood stage. Accordingly, we selected a sample of university students who were nearing the end of their academic careers and about to enter the labor market.

The aim of the study was to identify a neurophysiological method by which it is possible to measure the relational bond between coach and coachee and to evaluate how this dimension changes across phases of a coaching session. To this end, we used a professional coach trained in the Core Coaching approach who helped some university students in their school-to-work transition. The use of neurophysiological methods and the measurement of the relationship are particularly important for young people during life and the school-to-work transition. As coaching allows people to identify goals, develop skills, and use instruments to cope with life’s changes and difficulties in general, it is also important to verify their cognitive and emotional states and activations. In this sense, neuroanalysis is a key element that allows for deepening emotional reactions and guides the coachee in planning and designing the best path for the coachee.

As previously stated, each Core Coaching session was based on three consequential phases: in the first, contact between coach and coachee takes place and a relationship is established. Moreover, the main theme of the coaching intervention is identified. In the second phase, the coachee, with the support of the coach, starts on an inner journey in which he/she begins to acquire greater and deeper self-awareness. To do this, the coachee is more focused on himself/herself than on the relationship with the coach. In the final phase, the coachee has reached a new state of clarity and self-awareness that leads him/her to experience a particular state of well-being. We assume that the emotional characteristics (i.e., valence, arousal, and engagement) of the coach-coachee relational bond vary during the three phases and that these affective changes can be measured by neurophysiological indices such as AWI (valence) [162,163,164], BAR and SCL (arousal) [114,166], and BATR (engagement) [168]. In the neuroscience research paradigm, it is common practice to simultaneously adopt different instruments [113] and different metrics within each instrument [133] to measure subjects’ reactions in order to gain a greater understanding of the phenomena being studied. Furthermore, by means of the DTW technique, each of these emotional indices, recorded separately for coach and coachee, will be converted to express the degree of relational similarity. Based on these premises, the following research hypotheses were examined:

**Hypothesis** **1 (H1).** *We expect a statistically significant difference at the level of the relational dimension between the first and second phases. In particular, the values of emotional valence (AWI), arousal (BAR and SCL), and engagement (BATR) will be greater in the first phase than in the second.*

The first phase serves to involve the coachee in building the relationship and trust, as already observed in other studies [180]. In this phase, as pointed out by Richter et al. [181], it is indeed crucial to build a good relational quality as well as define the goals of intervention. In the second phase of Core Coaching, the coachee, under the guidance of the coach, focuses on his/her negative emotions and attitudes to be resolved as they may hinder the achievement of goals [182]. As mentioned in other approaches, the aim of this phase is to promote a cognitive reframe in the coachee [181]. Therefore, we expect that in the second phase, there will be a decrease in relational similarity between coach and coachee.

**Hypothesis** **2 (H2).** *We expect a statistically significant difference at the level of the relational dimension between the first and third phases. In particular, the values of emotional valence (AWI), arousal (BAR and SCL), and engagement (BATR) will be greater in the third phase than in the first.*

As mentioned in the previous lines, the first phase concerns the building of a good relationship between coach and coachee [181]. Since every coaching intervention has the ultimate goal of leading to an improvement in the coachee’s well-being and ability to cope with his/her difficulties, which are some of the outcomes of coaching programs [183], we expect the relational similarity in the third phase to be higher than in the first.

**Hypothesis** **3 (H3).** *We expect a statistically significant difference at the level of the relational dimension between the second and third phases. In particular, the values of emotional valence (AWI), arousal (BAR and SCL), and engagement (BATR) will be greater in the third phase than in the second.*

Consistent with the above, the second phase is based on focusing on the emotions and attitudes that hinder the achievement of the goals [181] identified in the first phase [182], and the third phase is when these mental obstacles are resolved [183]. Therefore, we can expect the relational similarity of the third phase to be higher than the second.

## 2. Materials and Methods

The Core Coaching approach was explored during the research carried out in the BrainLab Neuromarketing Laboratory at IULM University in Milan (Italy). All participants were university graduate students approaching work placement. The coach’s task was to understand the students’ future goals, ambitions, and moods. Each coaching session was videotaped using two webcams (LifeCam Studio by Microsoft, Inc., Redmond, WA, USA).

Before starting the research, participants received instructions about the instrumentation and the experiment procedure, as well as information on the voluntary nature of their participation in the study without any reward, and the anonymity of their data. They were also informed of the use of the data for research and study purposes. The study was conducted in line with the Helsinki Declaration [184] as well as the data protection regulations of Italy. There was no medical treatment or other procedure that could cause discomfort to participants; therefore, no additional ethical approval was necessary. Before starting the experiment, participants signed an informed consent form to accept their participation in the study and ensure anonymity.

### 2.1. Study Population and Experimental Design

Participants in the pilot study were 16 students (8 males and 8 females, M = 24 years; SD = 0.96; range 20–26) and were selected on the basis of criteria to ensure homogeneity of the sample. In this respect, the sample was based on students from the same degree course (i.e., “Marketing, Consumption and Communication”) who voluntarily decided to participate in the transition from university to work. To control for possible sources of gender bias, the male and female groups did not differ in terms of age, as shown by the Welch’s *t*-test (males: M = 23.5, SD = 1.73; females: M = 23, SD = 1.20, t(1) = 0.66, *p* = 0.521). This sample choice corresponded to a convenience (or incidental) homogeneous strategy [185].

A sensitivity analysis performed with G*Power v.3.1.97 [186] under standard conditions (α = 0.05, 1 − β = 0.95, ⍴ = 0.5, 𝜀 = 1) revealed that the minimum detectable effect size would have f = 0.42—interpreted as “big” [187]. Unfortunately, the length of the experiment did not allow us to increase the sample size.

**Figure 1:**
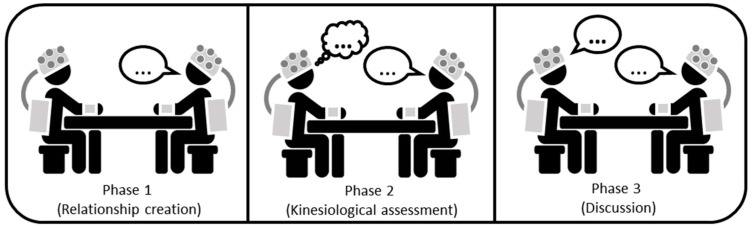
Schematic description of the experimental procedures underlying the three phases.

The coaching sessions made use of a professional coach practicing the Core Coaching approach, consisting of a face-to-face dialogue where the coach and coachee are positioned on a comfortable armchair. The sessions were always conducted by the same coach, i.e., a 55-year-old woman with a degree in business and economics who acquired the title of Professional Certified Coach (PCC) from the International Coach Federation (ICF) as an executive and team coach. The sessions were approximately 90 min long and consisted of three main phases. In phase 1, the relationship between coach and coachee is created. In phase 2, the coach assesses the road to reaching the objectives using kinesiologic tests. In phase 3, the coachee discusses his/her feelings after the session. Each phase was evaluated through instrumentation usually employed in neuroscience, such as an EEG headset and skin conductance. A schematic description of the experimental procedures underlying the three phases is provided in Figure 1.

#### Experimental Setting

Each research session took place in a room adjacent to the laboratory. It was purposefully decided to conduct the sessions in a neutral environment and not in the laboratory in order not to make the participants feel under pressure. Before starting with the research, the subjects were, of course, wired with all the instruments by the laboratory staff and made comfortable.

### 2.2. Instruments

The EEG was recorded using a 14-channel Epoc (Emotiv Inc., San Francisco, CA, USA) device, with a sample frequency of 128 Hz and a resolution of 14 bits. The device was in-house modified in order to improve its signal quality and mechanical stability [188]. The original water-based electrodes were replaced with gel-based Sn electrodes embedded in a medical-grade EEG cap (Taomed s.r.l.). The active electrodes did not change locations (still at Fp1, F7, F3, A1, T3, C3, T5, P3, O1, Fp2, F8, F4, A2, T4, C4, T6, P4, and O2 sites), while the reference and ground electrodes were replaced with two Sn ear clips attached to the left and right earlobes.

The SC signal was recorded using a Shimmer GSR+ (Shimmer Sensing LLC, Boston, MA, USA) with a sample frequency of 128 Hz and a resolution of 12 bits. According to the literature [189], the SC signal was recorded using the constant-voltage mode (0.5 V) from 2 Ag/AgCl electrodes placed on the index and ring fingers of the non-dominant hand.

Coach and coachee recordings were synchronized using their UTC timestamps, and 3 events corresponding to the 3 session phases (phase 1, phase 2, and phase 3) were manually placed by looking at the video recordings.

#### 2.2.1. EEG Processing

EEG signals were pre-processed using Matlab v. 2019a (The Mathworks, Inc., Natick, MA, USA) and the EEGLab toolbox [190] according to the following steps:

- Band-pass filter between 2 and 48 Hz (−6 dB cut-off frequencies at 1 and 49 Hz);
- Notch filter at both 50 and 100 Hz in order to reduce the powerline noise;
- Rejection of extreme sample points using an amplitude threshold (±100 μV) and a gradient threshold (±10 μV/ms).
- ICA (Independent Component Analysis) decomposition using the SOBI algorithm exhibits the best performance with respect to the majority of artifact types [191];
- Classification of ICs (Independent Components) using the 7-class neural-network classifier ICLabel [192]. That gives for each IC the class probability related to “brain”, “muscle”, “eye”, “hearth”, “line noise”, “channel noise”, and “other” classes.
- Identification of not-artifactual ICs using the following decision rule:

Pr{“brain”} > 0.70

OR

Pr{“brain”} ≥ 0.50 AND Pr{“brain”} + Pr{“other”} ≥ 0.70

- Rejection of other (i.e., artifactual) ICs and back-projection to the original sensor space;
- Epoching according to the session phases.

Because of the rejection of extreme points, homologous epochs related to each couple can have different lengths.

From each epoch, the following time-varying metrics (TVM) were computed:

1. Beta over alpha ratio (BAR);
2. Beta over alpha + theta ratio (BATR);
3. Approach-Withdrawal Index (AWI).

In order to compute the metrics, various EEG channels were filtered into different bands, and their instant power was computed. Given an individual alpha frequency (IAF) conventionally set at 10 Hz, the Theta (ϑ), Alpha (α), and Beta (β) bands are defined as: ϑ = [IAF-6; IAF-2], α = [IAF-2; IAF + 2], and β = [IAF + 2; IAF + 16] [193]. Instead of squaring the filtered signal, we applied a time-frequency approach: from each channel, we estimated the spectrogram using a Short-time Fourier transform (STFT) with a 1 s long hamming window and 50% overlap. Spectral bins corresponding to the selected band (either ϑ, α, or β) were summed (obtaining the channel instant power), and selected channels were averaged together (obtaining the group instant power). Finally, a logarithm transformation was applied in order to mitigate the skewness of the power values [194]. Because of the window’s length and the overlapping percentage of the STFT, the metrics had a temporal resolution of 0.5 s.

BAR is obtained as the ratio between the alpha and beta group powers, considering all the channels. Likewise, BATR is the ratio between beta group power and the sum of alpha and theta group powers. BAR has been previously associated with emotional arousal [166], while BATR has been previously adopted as the Engagement Index [168].

AWI is obtained as the difference between α-left and α-right group powers, considering as the left group the left frontal electrodes (Fp1, F7, F3) and as the right group the right frontal electrodes (Fp2, F8, F4). It has been previously associated with the approach-withdrawal behavior, which mostly correlates with emotional valence. In particular, higher activation in the left or right frontal hemisphere is associated with positive and negative emotional valence, respectively [162,163,164,165].

All metrics were z-score transformed according to the mean value and the standard deviation of the entire signal [195]

x (t) = [x′ (t) − mx]/sx

where x (t) is the z-score transformed metric, mx and sx are, respectively, its mean value and standard deviation, and x′ (t) is the original (raw) metric.

For each phase and each couple, we computed the similarity between coach and coachee TVMs using the Dynamic Time Warping (DTW) technique. DTW is a shape-based distance measure very popular in the literature of time series distance measures [174] that finds the best similarity of two signals by applying a temporal warping (i.e., contraction or dilatation). The temporal stretching allows for an optimal path (the non-linear mapping of the most common local features) while minimizing the distance of the wrapped signals. The application of DWT in EEG signal processing is quite usual, and its feasibility to identify EEG waveforms has been previously demonstrated [175]. In the present work, the chosen DTW distance was the Euclidean distance, and the similarity measure (TVM similarity, S-TVM) was its inverse.

#### 2.2.2. SC Processing

Similarly to the EEG, SC data were processed using Matlab. Since the maximum bandwidth of the SC signal is about 0.37 Hz [196], the SC signal was first undersampled to 1 Hz, and then an artifact correction algorithm was applied. SC data were first band-pass filtered using a zero-phase 4^th^-order FIR filter (0.001~0.35 Hz). Then, a threshold for SC extreme values (0.05~60 μS) and extreme rate of changes (±8 μS/s) was used in order to detect artifacts. Once identified, artifactual points were replaced by a linear interpolation [197].

From artifact-corrected SC, its tonic Skin Conductance Level (SCL), by means of the cvxEDA algorithm [198].

SCL is generally considered a robust estimation of emotional arousal with a low sensitivity to emotional valence [167]. SCL values have been shown to increase according to specific discrete emotions such as anger, anxiety, and amusement [199], while several features derived from SCL (e.g., mean and maximum value, peak-to-peak value, duration) have been used to train classification models [200,201].

In a similar fashion to EEG-related TVMs, the SCL TVM was epoched, and z-score transformed; then, the s-TVM was computed.

## 3. Results

### 3.1. Final Sample

After the processing steps, some subjects were rejected due to excessive noise or for missing either the EEG or the SC recordings. The final population of the experiment consisted of 14 subjects with EEG data (7 males, mean age 23.43 ± 1.50, range 20–26 years) and 7 with SC recordings (3 males, mean age 22.57 ± 1.51, range 20–25 years). Since Shapiro–Wilk revealed a lack of normality for the male group (W = 0.75, *p* < 0.001), SC data were analyzed through the Mann–Whitney test, while EEG data were analyzed through the Welch’s *t*-test. We did not find any significant difference in mean age between males and females for both the SC (males: M = 23, SD = 1.41; females: M = 22, SD = 1.73; W = 7, *p* = 0.853) and the EEG (males: M = 23.71, SD = 1.11; females: M = 23.14, SD = 1.86; t(1) = 0.70, *p* = 0.50).

#### 3.1.1. Statistical Analysis

Statistical analyses were performed using JASP v. 0.17.1, an open-source R-based statistical software package [202]. Since Mauchly’s test revealed the lack of sphericity (χ^2^(2) = 29.412, *p* < 0.001 for the EEG and χ^2^(2) = 29.040, *p* < 0.01 for the SC) and due to the small sample size [203], either non-parametric tests or parametric tests on the rank-transformed data [204] were applied. In order to analyze the effects of the sample variables, we tested each rank transformed TVM similarity through a mixed model ANOVA, considering the phase as within the subject factor, the gender as between the subject factor, and the age as a covariate. Then, for each TVM similarity, we applied Friedman’s test, considering the phase as a within-subject factor. Conover’s tests with Holm’s correction were chosen to perform post hoc comparisons.

#### 3.1.2. EEG and SC Results

Significant differences were found for all EEG-related s-TVMs: χ^2^(2) = 15.571, *p* < 0.001 for AWI, χ^2^(2) = 16, *p* < 0.001 for BAR, and χ^2^(2) = 13.857, *p* < 0.001 for BATR. For all the EEG-related s-TVMs, post hoc analysis confirmed significant (*p* < 0.05) differences between phases 1 and 2 and between phases 2 and 3, but not between phases 1 and 3. No differences were found for the S-TVM of the SCL, χ^2^(2) = 5.429, *p* = 0.066. The mixed model ANOVAs did not show any significant main effect of both the gender and the age for AWI (gender: F(1,11) = 2.303, *p* = 0.157; age: F(1,11) = 0.002, *p* = 0.962), BAR (gender: F(1,11) = 2.232, *p* = 0.163; age: F(1,11) = 0.019, *p* = 0.894), BATR (gender: F(1,11) = 2.778, *p* = 0.124; age: F(1,11) = 0.005, *p* = 0.946), and SCL (gender: F(1,4) = 0.960, *p* = 0.389; age: F(1,4) = 1.472, *p* = 0.292).

The following Table 1 reports the descriptive statistics (expressed as mean ± standard deviation) for the EEG- and SC-related s-TVMs and the significant pairwise comparisons (marked by * and ^†^).

## 4. Discussion

Our pilot study contributes to expanding the recent field of research concerning the application of neurophysiological techniques to coaching activities, also known as neurocoaching, by investigating a dimension not yet explored by previous studies and consisting of the relational bonding between coach and coachee. In order to achieve this purpose, a sample of university students participated in a coaching program conducted by a professional coach with the aim of facilitating their school-to-work transition. The coach, trained in the Core Coaching approach, structured each session according to three consequential phases. During the sessions, neurophysiological activity was recorded on both the coach and the coachee continuously and simultaneously. The affective dimension of relationships was calculated by processing the temporal signals of some specific emotional neurophysiological indices previously validated in the neuroscientific literature. These indices concerned emotional valence (AWI) [162,163,164,165], arousal (BAR and SCL) [166,167], and engagement (BATR) [168]. The relational component of these emotional indices was obtained by estimating the similarity of each pair of temporal signals relative to coach and coachee using the technique of Dynamic Time Warping [174,175]. Since the emotional-relational component of a helping relationship, as coaching is, changes over time, we expected it to be different during the three phases of each session. The study showed that the level of relational bonding between coach and coachee in the first and third phases is greater than in the second, whereas no difference emerged between the first and third phases. These results shed light on the fact that the nature of the coaching phases, which is based on the different techniques utilized by the coach, is the cause of relational differences between coach and coachee that can be estimated through neurophysiological techniques. Additionally, the absence of the gender effect on coachees supports the interpretation that the results observed in the research depend on the neurocoaching approach used.

According to the first hypothesis (H1), which expected a higher level of emotional valence (AWI), arousal (BAR and SCL), and engagement (BATR) in the first phase compared to the second phase, the emotional indicators of the relationship showed lower values in the second phase of the session than in the first. This hypothesis was partially confirmed by AWI, BAR, and BATR, but not with regard to SCL. These results are consistent with our expectations of the relational meaning of the first and second stages; indeed, the process of relationship development and mutual trust that occurs in the first stage may result in greater positive emotional value (AWI), arousal (BAR), and engagement. The fact that BAR is statistically significant and not SCL raises an important element: BAR, indeed, is associated with cognitive arousal [166], whereas SCL is an indicator of arousal that tends to grow when there are factors such as anxiety, anger, and enjoyment [167]. Considering the nature of the first phase in which the two subjects are at the beginning of their relationship and are still regulating it through the “ice-break”, it would be expected that the level of the cognitive side and of engagement are higher than the level of emotional intensity.

This comparison and results between the first and second phases are also in line with the specificity of the second phase, in which the coachee is more self-focused, which leads to less involvement in the bond with the coach.

As for the second hypothesis (H2), which expected a higher level of emotional valence (AWI), arousal (BAR and SCL), and engagement (BATR) in the third phase than in the first, results did not show any significance on the indices, thus H2 was rejected. Our interpretation of these results is related to the number of sessions experienced by the coachees. In our study, each coachee only participated in one session, but coaching programs can be structured over several sessions over time, e.g., from 1 up to 7 for the same coachee [203]. In this regard, studies have shown that increasing the number of sessions positively influences the relational quality between coach and coachee, thus improving the effectiveness of coaching interventions [205,206]. Since the third phase of the coaching session used in our research is the one in which a relational improvement between coach and coachee should have occurred compared to the first phase, it is possible that this result would have been achieved if the number of sessions per coachee had been greater. This standpoint is in line with studies suggesting that the dialogue between coach and coachee is influenced by co-creation [7], and in a short time it would not be possible to establish a deep relationship [24]. This aspect could be investigated by future studies that would consider longitudinal methodology to test the effect of deep knowledge and relationships on coach–coachee dynamics.

Finally, the third hypothesis (H3), which expected a higher level of emotional valence (AWI), arousal (BAR and SCL), and engagement (BATR) in the third phase than in the second phase, was also partly supported by the cortical indices. In this regard, our results showed higher values of AWI, BAR, and BATR in the third phase than in the second. In the last phase, the coachee has just emerged from the second one, characterized by an intense moment of introspection with a consequent decrease in relational bonding, and tunes back into the relationship with the coach in the light of a sense of trust. Again, considering the meaning of SCL [167], anxiety, anger, or high enjoyment in a phase in which the coachee is focused on the understanding of the thoughts that emerged during the coaching session are less expected than a cognitive elaboration.

In general (i.e., considering the emotional fluctuations along the phases), our results are consistent with the literature. Considering AWI (emotional valence), studies showed that subjects who display greater approach behavior and social competencies have a higher frontal asymmetry in the alpha band in the left hemisphere. Conversely, those with greater avoidance behavior and social wariness exhibit a higher frontal alpha band asymmetry in the right hemisphere [207,208]. Indeed, previous research has shown that increased activation of the frontal lobe of the right or left hemisphere is correlated with positive and negative emotional valence, respectively [162]. Moreover, research on parent–infant interactions using the Still Face Paradigm (corresponding to the absence of social cooperation on the part of the caregiver) has shown that dyads with a more responsive mother (i.e., cooperating in the interaction) exhibit higher alpha levels in the left region than dyads with a less responsive mother, which show higher alpha values in the right one [165]. In addition, evidence of the correlation between AWI and emotional valence in coaching was demonstrated by Jacks et al. [115]. Two further thoughts concern the coachee. The first is related to the fact that in the second phase, the coachee experiences and deepens knowledge of those problematic and negative thoughts that will then be resolved in the third phase. A recent study by Millis et al. [209] would support this interpretation, as the researchers observed correlations of the alpha rhythm in the right frontal hemisphere with negative thoughts about oneself, more reliance on others, and less cooperation. In this regard, we cannot exclude the possibility that the perception of negative thoughts may elicit a circular causal relationship with negative emotions, in which these constructs reinforce each other. The second considers David Rock’s description of the four phases that are part of the insight process, according to whom the last two (called “illumination” and “motivation”, respectively) are characterized by an increase in energy resulting from the awareness of new ideas and the motivation to put them into practice [210]. A study that investigated AWI during cognitive reappraisal showed a greater activation of the left prefrontal area in relation to the generation of new ideas [211]. This result would be consistent with the increase in AWI we observed in the last phase of the session compared to the second, in which such new ideas were not yet generated. The amount of physiological arousal (SC) between patient and therapist during the therapeutic process has been observed to be associated with states of empathic attunement [169,170,171,172] and correlated with treatment outcome [212]. In contrast to previous studies, we confirmed the role of arousal in relational bonding by obtaining statistically significant results through the electroencephalographic instrument (i.e., BAR) but not by means of the skin conductance (SCL). With reference to BATR, its social application occurred on the topic of social expectations (i.e., stereotypes) [173]. In our study, BATR values follow the same trend already observed in emotional valence (AWI) and arousal (BATR) across phases, hence supporting its appropriateness in measuring social engagement.

The research offers several theoretical insights. It introduces a way of operationalizing a construct that has so far not been investigated by neurocoaching research but which the wider literature has shown to have a relationship with coaching effectiveness, namely the relational dimension that binds coach and coachee [18,19]. In this sense, our study is the first attempt to introduce an objective methodology to measure the neurophysiological correlates of the relationship, in the hope that further studies can be conducted in the future. This relational construct was elaborated through a technique of estimating the similarity of temporal signals, measured on both coach and coachee, related to each of the emotional metrics used in the study (AWI, BAR, SCL, and BATR). In addition, regardless of the type of approach, coaching sessions are usually divided into different phases. The relational similarity technique used here can make it possible to identify relational trends across these phases in order to evaluate how they contribute to the general effectiveness of a coaching intervention. Moreover, the investigation we conducted may suggest a way to compare different approaches to coaching from the point of view of the impact exerted by the relational dimension. Furthermore, the research represents the first attempt to measure with neuroscientific techniques the approach called Core Coaching [45], thus expanding the theoretical frameworks of coaching that have received neurophysiological evidence in the literature.

Managerially, the study results show some actionable implications. The findings suggest the possibility of integrating the Core Coaching approach with a neuroscientific methodological support aimed at providing an objective estimate of the effectiveness of interventions within organizational contexts but also in offering coaching sessions to students deciding their future life, work, and school to make them more aware of their decisions and resilient about their future. In fact, this study highlights the adequacy of neurophysiological techniques in helping the life transition of young students approaching the world of work for the first time. The issue of life transition is relevant and has to be monitored in light of the development of strategies that could help students manage their transitions and their future. As mentioned, indeed, transitions may be very stressful [49,213], as it implies a high effort in changing behaviors, lifestyle, and choices [59,60,62], and one of the indicators of the success of life transitions can be the individual’s satisfaction and positive evaluation or interpretation of that moment [65,72]. In this view, knowing the coach’s effectiveness in the different phases of the coachee evaluation is crucial. In this sense, neuroanalysis could help to deepen emotional reactions and the cognitive side that can guide the coachee in designing the best path for the coachee. The emotional and cognitive reactions are, indeed, crucial elements for the coachee’s work: the set of feelings, thoughts, and behaviors that people use to manage difficult or worrying situations is a competence that emerges as crucial when life changes occur, such as work transitions, as it significantly influences well-being during the transitions themselves [214]. Therefore, the quality of the experience during life transition is relevant and should be monitored also during the coachee sessions in order to intervene, prevent negative experiences, and offer the coachee the right instrument at the right moment. This would be functional to guide the success of the sessions and to obtain a final evaluation of the transition experience, enhancing the sense of self-efficacy and giving the tools, knowledge, and ability to cope with life after the school-to-work transition. Moreover, helping individuals with coaching during the school-to-work transition can enhance their employability success [215]. Being aware of this interweaving and identifying the variables that contribute to the process can help people manage situations in a flexible way, considering a specific event with a higher quality of life over a long period of time.

As for organizational contexts, neurocoaching would be functional in enhancing well-being and activating those organizational cultures that are able to create positive organizational cultures that prevent burnout and discomfort [216]. Beyond the application of coaching to guide engagement, professional learning, development, and performance [28], preparing students before work could be functional in making their choices more effective and engaging, enhancing engagement and psychological well-being. Studying emotional reactions through neuroscience allows the professional coach to better guide and design sessions and interventions. This would have implications for both individuals and organizations. Individuals, indeed, would be guided in their level of well-being, preventing discomfort, monitoring the perception of the level of demands in the workplace [217], and placing neurocoaching as a resource in the workplace, thereby preventing the risk of workaholism [218]. This would be functional to better achieve goals and enhance self-efficacy [219], reinforcing organizational productivity and performance. Monitoring this aspect through neuroscientific tools and giving workers the instruments to face a demanding workplace would be useful to reduce the possibility of workaholism problems [220] or other emotional, cognitive, and physical discomfort for workers.

### Limits of the Study

This study is not without limitations, which provide avenues for further research. First, all coaching sessions were conducted by the same professional coach. The lack of gender differences in this sample is in line with previous studies [221,222], which showed that gender does not appear to play a crucial role in the matching process [223]. However, due to the complexity of gender matching in coaching sessions [221], further investigations with neuroscientific tools could deepen the issue.

Indeed, future studies could use different coaches, following the same coaching approach, in order to exclude the confounding variable of the coach as such. Second, in some cases, the EEG indices had high standard deviation scores. This issue can be solved by integrating, before starting the experiment, a resting-state baseline phase through which each metric can be standardized. Third, unlike other studies that used physiological arousal, we did not find statistical significance with regard to SCL. This result could be investigated in order to identify the reasons for it. Fourth, we found no statistically significant differences between the first and last phases. This result would require further research to ascertain whether, within a coaching program involving several sessions, it might be observable. Last, even though we utilized electroencephalographic devices for the research, the implementation of hyperscanning techniques [224] would allow a finer analysis (e.g., sample by sample) of the proposed S-TVMs in order to find possible tuning within each phase of the session.

Not-significant results could also be due to the lack of sensitivity of our experimental design, which allowed us to capture only a “big” effect size (f = 0.42). As previously noted, the length of the experiment limited our initial sample size to 16, and the rejection due to the artifact contaminations even lowered it; this almost certainly increased the minimum detectable effect size. A future confirmatory study involving a greater number of participants should be conducted. As suggested by an a-priori analysis performed using G*Power, keeping the number of repeated measurements constant (3) and under standard conditions (α = 0.05, 1 − β=0.95, ⍴ = 0.5, 𝜀 = 1), at least 43 subjects are required to capture a “medium” effect size (f = 0.25) [187].

Fifth, it must be noted that the building strategy of the sample, based on the convenience (or incidental) approach, has a well-known limitation: since it is based on a specific non-random sociodemographic group, the obtained results have limited generalizability to larger populations [225]. Despite the fact that the extension to a wider group of subjects was not within the scope of the present study, we must underline this restriction to avoid any misinterpretation of the results. Given these premises, we cannot, however, deem convenience sampling a fatal flaw since it is considered an acceptable approach at least in exploratory and pilot studies [225], and it was found to be very common in disciplines such as development science [226]. A future confirmatory study should add, as controlled factors, other variables (e.g., different degree programs and different professional coaches) to provide more accurate population estimates.

## 5. Conclusions

In this pilot study with an exploratory aim, we introduced a novel method to measure the relational dimension in coaching interventions by using some techniques belonging to the neuroscientific research paradigm. The study does not aim to generalize results but tries to suggest a view on the coaching session using different tools from traditional research through a methodology and indicators belonging to the neuroscientific area [114,162,163,164,165,166,167,168,169,170,171,172,173,174,175]. In fact, the scientific literature has previously verified that the relationship between coach and coachee has an impact on the effectiveness of the coaching intervention. For this purpose, we used electroencephalographic and skin conductance devices to estimate the degree of similarity of temporal signals related to certain neurophysiological indices associated with emotions that have been extensively validated by neuroscientific research. These indices were measured continuously on both coach and coachee during a series of coaching sessions aimed at facilitating the life transition of a group of university students into the world of work. We found that changes in relationship levels during coaching sessions can be consistently estimated by measuring cortical electrical activity. These changes reflect the different levels of relational bonding between coach and coachee that occur during the phases of a coaching session. Future studies could confirm and extend our results using finer techniques, such as hyperscanning, and involving different coaches and approaches. Further studies should be conducted to analyze team coaching sessions and design thinking approaches by applying neuroscientific observations to relational bonds between all the participants due to the influence peer-to-peer interactions have on the final output. Finally, the study provides a useful method that can be implemented within coaching programs to assess the impact of the relational dimension and, therefore, their effectiveness. Coaching in organizations opens a new area of research because the relationship between coach and coachee is deeply influenced both by the peer-to-peer interactions in the teams and by the organizational culture. Change management processes are more and more conducted through team coaching methods, where each coachee becomes an actor in the process. Another area of research is design thinking, an approach to innovation and problem solving with several similarities to coaching: again, every stakeholder in the process is a change maker, and a specific neuroscientific analysis should be applied [172,227,228].

## Figures and Tables

**Table 1:** Descriptive statistics for the EEG- and SC-related s-TVMs expressed as mean ± standard deviation, with significant pairwise comparisons marked by * and ^†^.

Phase	AWI	BAR	BATR	SCL
1	0.0030 ± 0.0030 *	1.1560 ± 1.6850 *	0.8320 ± 0.9330 *	0.0070 ± 0.0130
2	0.0008 ± 0.0001 *^,†^	0.0860 ± 0.0360 *^,†^	0.0960 ± 0.0420 *^,†^	0.0002 ± 0.0002
3	0.0080 ± 0.0130 ^†^	10.2600 ± 22.2220 ^†^	3.8530 ± 5.4390 ^†^	0.0020 ± 0.0030

## Data Availability

The data presented in this study are available on request from the corresponding author.
